# Factors Associated with Uncomplicated Single-Session Stone-Free Status After Retrograde Intrarenal Surgery for Large Renal Stones

**DOI:** 10.3390/jcm15145530

**Published:** 2026-07-15

**Authors:** İbrahim Kartal, Ishak Günay, Halil İbrahim Ivelik, Şeref Çoşer, Mehmet Sevim, Okan Alkış, Şahinde Atlanoğlu, Bekir Aras

**Affiliations:** 1 Department of Urology, Faculty of Medicine, Kütahya Health Sciences University, Kütahya 43020, Türkiye; ishak.gunay@ksbu.edu.tr (I.G.); okan.alkis@ksbu.edu.tr (O.A.);; 2 Department of Urology, Faculty of Medicine, İzmir Bakırçay University, İzmir 35665, Türkiye; 3 Department of Urology, Konya City Hospital, Health Sciences University, Konya 42080, Türkiye; 4 Department of Radiology, Faculty of Medicine, Kütahya Health Sciences University, Kütahya 43020, Türkiye

**Keywords:** retrograde intrarenal surgery, nephrolithometry, stone-free rate, kidney calculi, CROES score, Ito’s nomogram, TOHO score, RUSS score

## Abstract

**Background:** To identify predictors of uncomplicated single-session stone-free (USSSF) outcomes after retrograde intrarenal surgery (RIRS) for large renal stones and to compare the performance of contemporary nephrolithometry systems. **Methods:** We retrospectively analyzed 158 patients who underwent RIRS for 20–40 mm renal stones. Demographic, stone-related, anatomical, and perioperative variables were recorded. R.I.R.S., RUSS, modified S-ReSC, CROES, Ito nomogram, TOHO, and STONE scores, as well as the renal parenchyma-to-renal volume ratio, were calculated from preoperative non-contrast computed tomography images. USSSF was defined as achievement of stone-free status or clinically insignificant residual fragments (CIRFs <4 mm) after a single RIRS session, without postoperative complications, retreatment, or auxiliary procedures. Logistic regression and receiver operating characteristic analyses were performed. **Results:** USSSF was achieved in 90 patients (56.9%). Lower stone burden, fewer stones, absence of hydronephrosis or abnormal renal anatomy, no previous intervention, and greater surgeon experience were associated with USSSF. On multivariable analysis, higher CROES and Ito’s nomogram scores independently predicted USSSF, whereas higher TOHO and RUSS scores were associated with lower success rates. Among all nephrolithometry systems, the CROES score and Ito’s nomogram demonstrated the highest discriminative ability (area under the receiver operating characteristic curve [AUC], 0.896 and 0.862, respectively). **Conclusions:** In patients undergoing RIRS for 20–40 mm renal stones, the CROES score and Ito’s nomogram were the strongest predictors of achieving USSSF. These scores may be useful for preoperative patient counselling and risk stratification in selected patients with complex renal stones.

## 1. Introduction

Kidney stone disease represents a substantial global health burden, with an increasing prevalence in both developed and developing countries. According to the European Association of Urology (EAU) guidelines, percutaneous nephrolithotomy (PCNL) is recommended as the first-line treatment for renal stones larger than 20 mm, whereas retrograde intrarenal surgery (RIRS) may be considered as an alternative in selected patients in whom percutaneous access is undesirable or carries increased risk [1].

Although stone-free rate remains the most commonly reported outcome after RIRS, it does not fully reflect procedural success in clinical practice. Postoperative complications, the need for auxiliary procedures, and the feasibility of achieving stone clearance within a single session are also highly relevant, particularly in patients with large renal stones. For this reason, evaluating uncomplicated single-session success (USSSF) may provide a more clinically meaningful assessment of RIRS outcomes than stone-free status alone.

Recent advances in flexible ureteroscope technology, laser lithotripsy systems, and the use of ureteral access sheaths have expanded the role of RIRS in the management of larger renal stones. Consequently, RIRS is increasingly being performed in selected patients with stones exceeding 20 mm, particularly when a less invasive treatment option is preferred or when PCNL may be associated with increased morbidity. However, treatment outcomes in this setting remain variable, and careful preoperative assessment is essential for appropriate patient selection and counselling [2,3,4].

Several nephrolithometry scoring systems have been developed to predict outcomes after endourological procedures, but most were designed for smaller stones, and their performance in patients with large renal stones remains uncertain [5,6,7,8].

Therefore, the aim of the present study was to evaluate the factors associated with achieving USSSF following RIRS in patients with large renal stones and to investigate the predictive value of commonly used scoring systems in this patient population.

## 2. Materials and Methods

This retrospective study was conducted at a tertiary referral center after obtaining approval from the local ethics committee (approval number: 2026/02-52). The study was performed in accordance with the ethical principles of the Declaration of Helsinki. Due to the retrospective design, the requirement for informed consent was waived.

The medical records of 831 patients who underwent RIRS between January 2022 and December 2025 were retrospectively reviewed. Patients were excluded if they had renal stones smaller than 20 mm, ureteral stones, missing data, loss to follow-up, chronic kidney disease, age <18 or >80 years, or renal stones larger than 4 cm, which are rarely managed with RIRS in our practice.

In accordance with EAU recommendations, patients with renal stones between 20 and 40 mm in cumulative diameter and a preoperative serum creatinine <1.65 mg/dL were included. Although PCNL is generally considered the standard treatment for renal stones >20 mm, RIRS was preferred in selected patients following a shared decision-making process between the patient and the treating surgeon. Factors influencing the selection of RIRS included patient preference, comorbidities, bleeding risk, unfavorable conditions for percutaneous access, and surgeon discretion. Patients with active urinary tract infection received appropriate antibiotic therapy and were operated on only after obtaining sterile urine culture results. After applying the inclusion and exclusion criteria, 158 patients were included in the final analysis.

Demographic and clinical characteristics were recorded, including age, sex, medical history, Charlson Comorbidity Index (CCI) and body mass index (BMI). Stone-related and renal anatomical parameters were also evaluated: stone size, stone volume, stone location, stone density (Hounsfield units, HU), presence of urinary tract anomalies, number of stones, presence of lower pole stones, infundibulopelvic angle (IPA), renal infundibular length (RIL), and hydronephrosis. Operative and postoperative variables included: preoperative ureteral stent placement, operative time, residual stone fragments, length of hospital stay, and postoperative complications. Operator experience was defined according to the cumulative number of RIRS procedures previously performed by the operating surgeon and was categorized as <50 or ≥50 cases.

All procedures were performed under general anesthesia in the lithotomy position. After ureteral evaluation with a semi-rigid ureteroscope, a guidewire was advanced and a ureteral access sheath was placed when feasible. Flexible ureteroscopy with holmium: YAG laser lithotripsy (35 W; Quanta System S.p.A., Samarate, Italy) was then performed using fragmentation or dusting at the surgeon’s discretion. Stone fragments were either left for spontaneous passage or retrieved with a basket as needed, and the collecting system was systematically inspected at the end of the procedure. A double-J stent was placed according to clinical indications and was usually removed within 2–4 weeks. All procedures were performed or supervised by three senior urologists with at least 10 years of experience. Postoperative complications were classified according to the Clavien–Dindo classification system [9].

All patients underwent non-contrast computed tomography (NCCT) with a multidetector CT scanner. Stone size, volume, and density were measured on axial, coronal, and sagittal images using standard abdominal window settings. Stone size was defined as the maximum axial diameter, and stone volume was calculated using the ellipsoid formula based on axial, coronal, and sagittal diameters. Stone burden for the CROES nephrolithometry score was calculated in mm^2^ using the following formula: 0.785 × maximum length × maximum width. In patients with multiple stones, individual stone volumes were calculated and summed to determine the total stone burden. The IPA was defined as the angle formed between the ureteropelvic axis and the axis passing through the center of the lower pole infundibulum. The renal infundibular length (RIL) was measured as the distance from the base of the lower calyx to the inferior margin of the renal pelvis. All measurements were initially performed by one urologist (I.K.) and subsequently reviewed by an experienced radiologist (Ş.A.). Discrepancies between evaluators were resolved by consensus to minimize measurement variability.

Each patient was evaluated according to several nephrolithometry scoring systems using preoperative NCCT images. The following scoring systems were calculated: Resorlu–Unsal Stone Score (RUSS), modified Seoul National University Renal Stone Complexity (S-ReSC) score, Clinical Research Office of the Endourological Society (CROES) nephrolithometry score, Ito’s nomogram, the TOHO score, R.I.R.S. score and the STONE score (Stone size, Tract length, Obstruction, Number of involved calyces, and Essence) [10,11,12,13,14,15,16]. Additionally, the renal parenchyma-to-renal volume ratio (RPRV) was calculated according to the method described by Zhen et al. [17].

Patients were categorized into two groups based on the primary study outcome. The primary outcome of the study was USSSF, defined as achievement of complete stone-free status or clinically insignificant residual fragments (CIRFs < 4 mm) after a single RIRS procedure, without postoperative complications, retreatment, or auxiliary surgical intervention. Residual fragments smaller than 4 mm were considered CIRFs according to commonly used endourological definitions and were included in the successful outcome group [18]. According to the findings of the NCCT performed at 1 month postoperatively, patients were classified as successful or unsuccessful based on the achievement of the defined outcome. NCCT was preferred for postoperative assessment because of its high sensitivity for detecting residual stone fragments.

### Statistical Analysis

All statistical analyses were performed using IBM SPSS Statistics for Windows, version 27.0 (IBM Corp., Armonk, NY, USA). A two-sided *p* value < 0.05 was considered statistically significant. The distribution of continuous variables was assessed using the Kolmogorov–Smirnov test. Continuous variables with normal distribution were expressed as mean ± standard deviation (SD), whereas non-normally distributed variables were presented as median and interquartile range (IQR). Categorical variables were summarized as frequencies and percentages.

Comparisons between groups according to USSSF were performed using the Student’s *t*-test or Mann–Whitney U test for continuous variables, depending on data distribution. Categorical variables were analyzed using the chi-square test or Fisher’s exact test, as appropriate.

To identify potential predictors associated with failure to achieve USSSF, univariate logistic regression analysis was initially performed. Variables with *p* < 0.10 in univariate analysis were subsequently included in the multivariable logistic regression model. Multivariable analysis was conducted using backward stepwise binary logistic regression to identify the most clinically relevant independent predictors while minimizing model overfitting, and the results were reported as odds ratios (ORs) with 95% confidence intervals (CIs).

Due to potential multicollinearity between nephrolithometry scores and individual stone-related variables, nephrolithometry scoring systems were evaluated in separate multivariable regression models. The goodness-of-fit of the final multivariable models was assessed using the Hosmer–Lemeshow test. Separate models were also preferred to reduce model complexity and the risk of overfitting given the sample size.

The predictive performance of the evaluated scoring systems was assessed using receiver operating characteristic (ROC) curve analysis. The area under the ROC curve (AUC) was calculated to determine the discriminative ability of each scoring system, and the optimal cut-off values were determined according to the maximum Youden index.

## 3. Results

A total of 158 patients met the inclusion criteria and were included in the final analysis, of whom 90 (56.9%) achieved USSSF, whereas 68 (43.1%) did not. Baseline demographic and clinical characteristics were generally comparable between the groups, with no significant differences in age, sex distribution, BMI, or renal function. However, patients who achieved USSSF more frequently had a CCI ≤ 2, lower stone burden, fewer stones, and less complex stone configuration than those who did not.

To improve the interpretation of the composite endpoint, complete stone-free status and clinically insignificant residual fragments (CIRFs) were analyzed separately. Of the 90 patients who achieved USSSF, 79 (50.0% of the entire cohort) were completely stone-free on postoperative NCCT. The remaining 11 patients had residual fragments <4 mm and were included in the successful outcome group according to the predefined study definition. Therefore, the complete stone-free rate was 50.0%, whereas the USSSF rate was 56.9%.

To further characterize the components of the composite endpoint, the reasons for failure to achieve USSSF were analyzed. Among the 68 patients classified as unsuccessful, residual stone fragments > 4 mm were present in 65 patients (95.6%). The remaining 3 patients were completely stone-free on postoperative NCCT but were classified as unsuccessful because of postoperative complications. Retreatment was required in 22 patients, whereas auxiliary procedures were performed in 16 patients, including Extracorporeal Shock Wave Lithotripsy in 12 patients and PCNL in 4 patients. These findings indicate that failure to achieve USSSF was predominantly driven by residual stone burden rather than complications alone.

Postoperative complications occurred in 10 patients (6.3%). According to the Clavien–Dindo classification, 3 complications were classified as Grade I (two cases of transient hematuria and one case of self-limiting nausea/vomiting), 5 as Grade II (febrile urinary tract infection requiring antibiotic treatment), 1 as Grade IIIa (double-J stent migration requiring stent replacement under local anesthesia), and 1 as Grade IIIb (ureteroscopy performed for symptomatic steinstrasse). No Grade IV or Grade V complications were observed.

Stone-related and anatomical factors showed marked differences between the groups. Patients in the USSSF group had significantly smaller stone volume, fewer stones, and lower rates of abnormal renal anatomy and hydronephrosis. Nephrolithometry scores also differed substantially: the USSSF group exhibited higher median CROES and Ito’s scores, whereas TOHO, RUSS, and STONE scores were significantly higher in the non-USSSF group. Renal parenchymal volume and the parenchyma-to-renal volume ratio (RPRV) were greater in patients who achieved USSSF (Table 1).

On univariate logistic regression analysis, several variables were associated with failure to achieve USSSF. Higher CCI, larger stone volume, greater number of stones, abnormal renal anatomy, previous surgical intervention, and the presence of hydronephrosis were all significantly associated with unsuccessful outcome. Among the nephrolithometry scoring systems, higher R.I.R.S., RUSS, modified S-ReSC, TOHO, and STONE scores were associated with a lower likelihood of USSSF, whereas higher CROES and Ito’s scores were associated with a higher likelihood of USSSF. RPRV also showed a trend toward association with USSSF on univariate analysis (Table 2).

Because of potential multicollinearity, each nephrolithometry score was evaluated in a separate multivariable model adjusted for stone volume, number of stones, previous intervention, and operator experience. In these adjusted analyses, higher Ito’s nomogram and CROES scores were independently associated with a higher likelihood of achieving USSSF, whereas higher TOHO and RUSS scores remained independently associated with a lower likelihood of USSSF. R.I.R.S. and STONE scores showed similar trends but with weaker and less consistent associations after adjustment. Modified S-ReSC did not retain a significant association with USSSF in the adjusted models (Table 3).

**Figure 1:**
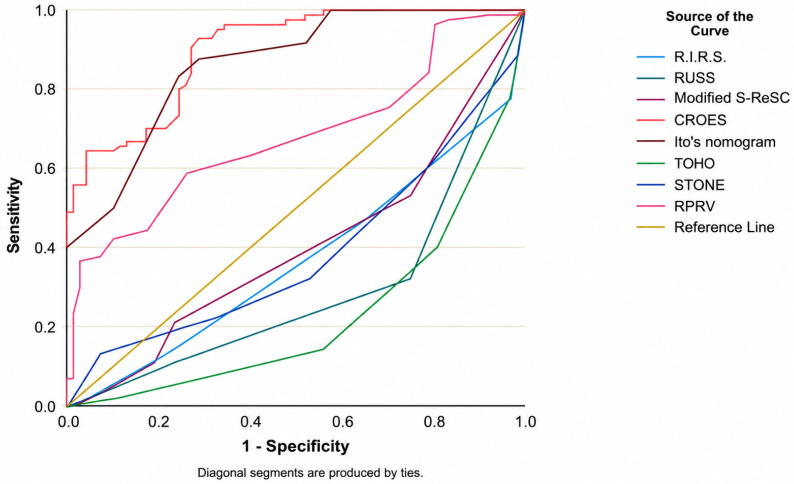
Receiver Operating Characteristic Curves of Nephrolithometry Scoring Systems and RPRV for Predicting Uncomplicated Single-Session Stone-Free Status.

ROC curve analysis was performed to further assess the predictive performance of the evaluated nephrolithometry systems and RPRV. Among all models, the CROES score showed the highest discriminative ability for predicting uncomplicated single-session stone-free status (AUC 0.896), followed by the Ito nomogram (AUC 0.862). RPRV demonstrated only modest predictive performance. In contrast, TOHO, RUSS, R.I.R.S., modified S-ReSC, and STONE scores showed inverse associations with USSSF, reflecting the lower likelihood of successful single-session outcomes in patients with increasing stone complexity. Therefore, AUC values below 0.5 in these systems should be interpreted as reflecting an inverse association with USSSF rather than inadequate predictive performance, since higher scores indicate greater stone complexity and a lower probability of successful outcomes. The ROC curves of all scoring systems and RPRV are presented in Figure 1, and the corresponding AUC values are summarized in Table 4.

## 4. Discussion

In the present study, we evaluated factors associated with USSSF after RIRS in patients with renal stones measuring 20–40 mm and compared the predictive performance of several nephrolithometry systems. USSSF was achieved in 57% of patients, indicating that although RIRS can be effective in selected large renal stones, single-session success without complications cannot be assumed. Therefore, careful preoperative risk stratification remains clinically important in this patient group. Unlike conventional analyses based solely on stone-free status, the present study focused on a composite endpoint that captures both treatment success (defined as complete stone-free status or clinically insignificant residual fragments <4 mm) and perioperative safety.

Consistent with previous reports, higher stone burden and multiplicity, abnormal renal anatomy, previous interventions, and hydronephrosis were associated with lower USSSF rates [19,20]. These factors may limit intrarenal access, prolong operative time, and increase the likelihood of residual fragments. Operator experience also had a clear effect on outcome, which is particularly relevant in large stones where RIRS may require more complex intraoperative decision-making. For this reason, operator experience was included in the adjusted models. However, the observed association should be interpreted with caution, as it may reflect not only technical proficiency but also learning-curve effects and temporal changes in patient selection throughout the study period. Similar observations have been reported in large endourological series, where higher procedural volume was associated with improved outcomes and lower rates of adverse events following ureteroscopic stone surgery [21]. Therefore, the observed effect cannot be attributed solely to technical expertise.

Among the evaluated scoring systems, Ito’s nomogram and the CROES score showed the strongest association with USSSF. One possible explanation is that these tools incorporate a broader range of clinically relevant variables beyond stone location alone. Ito’s nomogram is particularly relevant for RIRS because it includes factors such as surgeon experience, stone number, hydronephrosis, and lower-pole localization. Similarly, the CROES score incorporates stone burden and key clinical parameters that may influence the probability of complete stone clearance. In the present cohort, both scores remained independently associated with USSSF and demonstrated strong discriminative performance on ROC analysis.

Our findings should be interpreted in light of recent studies evaluating RIRS outcomes in large renal stones. Alma et al. evaluated patients with stones larger than 20 mm and reported acceptable predictive performance for R.I.R.S., RUSS, and modified S-ReSC scores, with AUC values around 0.73–0.75 [22]. In contrast, Polat et al. reported that existing scoring systems, including RUSS, modified S-ReSC, R.I.R.S., and Ito’s nomogram, had limited predictive ability in 2–4 cm stones, whereas their newly developed model performed better [23]. The stronger performance of Ito’s nomogram and CROES score in our cohort may be related to differences in patient selection, endpoint definition, imaging assessment, and the inclusion of uncomplicated single-session success rather than stone-free status alone. In another comparative study, Özbek et al. reported that although the R.I.R.S. scoring system showed relatively good performance for predicting stone-free status, none of the evaluated scoring systems were independently associated with postoperative complications [24]. In the present study, the use of USSSF as the primary endpoint may therefore better reflect real-world clinical outcomes after RIRS.

By contrast, higher TOHO, RUSS, STONE, and R.I.R.S. scores mainly reflected increasing case complexity and were associated with lower USSSF rates. This inverse relationship likely reflects greater procedural complexity in patients with higher nephrolithometry scores. Consequently, ROC analyses performed using USSSF as the outcome naturally result in AUC values below 0.5 for these scoring systems. Therefore, these findings should be interpreted as an inverse relationship with USSSF rather than as a complete lack of predictive value. Similarly, a recent systematic review and meta-analysis comparing contemporary RIRS scoring systems concluded that no currently available nephrolithometry model demonstrated clearly superior or consistently high predictive performance across studies [25].

Recently, interest has increased in renal parenchymal parameters as potential predictors of stone-free outcomes after RIRS. In a recent study, Zhen et al. introduced the ratio of renal parenchymal volume to renal volume (RPRV) and reported that lower RPRV values were associated with residual stones after RIRS, particularly in patients with 2–2.9 cm stones [17]. In our cohort, RPRV demonstrated only fair discriminative ability and remained inferior to Ito’s nomogram and the CROES score. Nevertheless, it may still provide complementary information regarding renal anatomy and drainage characteristics.

From a clinical perspective, these results suggest that RIRS may be a reasonable option for selected patients with 20–40 mm renal stones, particularly when nephrolithometry scores indicate favorable anatomy and stone burden. However, patients with high-complexity scores should be carefully counselled about the increased risk of residual fragments, the potential need for staged treatment, and alternative approaches such as PCNL. Importantly, this study was not designed to compare RIRS with PCNL, and the findings should therefore be interpreted within the context of patients selected for RIRS.

Several limitations of this study should be acknowledged. First, the retrospective single-center design may limit the generalizability of the findings and introduces an inherent risk of selection bias. In addition, the strong association observed for operator experience may partly reflect learning-curve effects and temporal changes in case selection over the study period. Although all measurements were reviewed by an experienced radiologist, interobserver variability was not formally analyzed. The relatively limited sample size may also have reduced the ability to fully evaluate the performance of nephrolithometry systems across different anatomical subgroups and stone distributions. Furthermore, the study focused on short-term perioperative outcomes, and long-term recurrence or reintervention rates were not assessed. Finally, the results may partly reflect the experience and case selection patterns of a high-volume tertiary referral center.

Despite these limitations, this study provides a direct comparison of several commonly used nephrolithometry scores within a single cohort of patients undergoing RIRS for large renal stones. The findings suggest that Ito’s nomogram and the CROES score may serve as practical tools for preoperative counselling and risk stratification in selected cases. Future prospective multicenter studies are warranted to further validate these results and to clarify the role of nephrolithometry systems in treatment selection and patient counselling for large renal stones.

## 5. Conclusions

In patients with 20–40 mm renal stones treated with RIRS, uncomplicated single-session stone-free status was achieved in just over half of the cases, underscoring the need for careful preoperative selection. Lower stone burden, absence of abnormal renal anatomy and hydronephrosis, no previous interventions, and greater operator experience favored this optimal outcome. Ito’s nomogram and the CROES score showed the best performance for predicting USSSF. Higher TOHO, RUSS, STONE, and R.I.R.S. scores mainly reflected greater procedural complexity and a lower probability of single-session success. These findings may help optimize patient selection and preoperative counseling for RIRS in large renal stones.

## Figures and Tables

**Table 1:** Demographic, Clinical, and Operative Characteristics According to Uncomplicated Single-Session Stone-Free Status.

Variables	Overall (n = 158)	Successful (n = 90, 56.9%)	Unsuccessful (n = 68, 43.1%)	*p*-Value
Age	43 (29–58.5)	43 (29–56.2)	45 (29.5–61.7)	0.444
Gender (Male/Female) n (%)	100 (63.3%)/58 (36.7%)	55 (61.1%)/35 (38.9%)	45 (66.2%)/23 (33.8%)	0.513
Body Mass Index (BMI)	27.2 (25.1–29.3)	27.2 (25.3–29.2)	27.3 (24.9–29.4)	0.919
CCI ≤ 2/> 2 n (%)	132 (83.5%)/26 (16.5%)	80 (88.9%)/10 (11.1%)	52 (76.5%)/16 (23.5%)	0.037
Maximum Stone Diameter (mm)	24.5 (22–30)	22.5 (21–24)	30.5 (26–34)	<0.001
Stone Area (mm^2^)	251.9 (189.5–366.5)	196.2 (169.5–224.5)	379 (329.7–435.6)	<0.001
Stone Volume (mm^3^)	1848.8 (1455.5–2400.5)	1521.9 (1284.6–1794.9)	2408.4 (2049.8–2847.7)	<0.001
Number of Stones	1 (1–3)	1 (1–2)	3 (1–4)	<0.001
Single vs. Multiple Stones n (%)	93 (58.9%)/65 (41.1%)	63 (70%)/27 (30%)	30 (44.1%)/38 (55.9%)	0.001
Single vs. Multiple Calyces n (%)	104 (65.8%)/54 (34.2%)	65 (72.2%)/25 (27.8%)	39 (57.4%)/29 (42.6%)	0.051
Stone Location Lower Pole Middle Pole Upper Pole Renal Pelvis Multicalyceal		23 (25.5%) 8 (8.8%) 7 (7.7%) 27 (30%) 25 (27%)	25 (36.7%) 2 (2.9%) 3 (4.4%) 9 (13.2%) 29 (42.6%)	0.190
Abnormal Renal Anatomy n (%)	16 (10.1%)	4 (4.4%)	12 (17.6%)	0.008
Stone Density (HU)	1105 (983.2–1192.5)	1077 (973–1183.5)	1158.5 (989.5–1197.2)	0.054
Previous Surgical Intervention n (%)	99 (62.7%)	47 (52.2%)	52 (76.5%)	0.002
Presence of Hydronephrosis n (%)	64 (40.5%)	29 (32.2%)	35 (51.5%)	0.015
Operation Time (min)	110 (100–120)	107.5 (100–120)	115 (105–120)	0.007
Operator Experience (<50 vs. ≥50 cases) n (%)	72 (45.6%)/86 (54.4%)	16 (17.8%)/74 (82.2%)	56 (82.4%)/12 (17.6%)	<0.001
Use of Access Sheath n (%)	146 (92.4%)	81 (90%)	65 (95.6%)	0.189
Preoperative DJS Placement n (%)	36 (22.8%)	26 (28.9%)	10 (14.7%)	0.035
Postoperative DJS Placement	152 (96.2%)	86 (95.6%)	66 (97.1%)	0.624
Length of Hospital Stay (days)	1 (1–1)	1 (1–1)	1 (1–2)	<0.001
R.I.R.S.	8 (7–8)	7 (7–8)	8 (7–8)	0.003
RUSS	2 (1–2)	1 (1–2)	2 (1.2–2)	<0.001
Modified S-ReSC	2 (1–2)	2 (1–2)	2 (1.2–2)	0.022
CROES	200 (164–281)	267 (198–299)	144 (89–199)	<0.001
Ito’s Nomogram	7 (3.7–9)	8.5 (7–12)	4 (0–6.5)	<0.001
TOHO	9 (8–10)	8 (8–9)	10 (9–10)	<0.001
STONE	11 (10–13)	11 (10–12)	12 (11–13)	0.012
Renal Volume (cm^3^)	193.6 (189.4–196.2)	193.4 (189.8–195.6)	194.5 (186.4–196.8)	0.525
Renal Parenchymal Volume	140.1 (135.3–145)	143.3 (138.4–147.9)	136.5 (133–139.7)	0.001
Parenchyma-to-Renal Volume Ratio (RPRV)	0.72 (0.69–0.75)	0.73 (0.69–0.78)	0.71 (0.69–0.73)	0.040
Infundibulopelvic Angle (IPA)	50 (25.7–58.2)	51 (25.7–59.2)	45 (25.2–57.5)	0.311
Renal Infundibular Length (RIL, mm)	22 (20.7–24)	22 (20–24)	23 (21–25)	0.630

**Table 2:** Univariable Logistic Regression Analysis for Predictors of Achievement of USSSF.

Variables	Univariate		
	OR	95% CI	*p*-Value
CCI ≤ 2 /> 2	0.410	0.17–0.96	0.041
Stone Volume	0.995	0.994–0.997	<0.001
Number of Stones	0.564	0.432–0.738	<0.001
Abnormal Renal Anatomy	0.217	0.067–0.707	0.011
Previous Surgical Intervention	0.336	0.168–0.675	0.002
Presence of Hydronephrosis	0.448	0.234–0.858	0.015
Operator Experience	21.583	9.458–49.253	< 0.001
Preoperative DJS Placement	2.356	1.047–5.303	0.038
R.I.R.S.	0.576	0.404–0.821	0.002
RUSS	0.367	0.231–0.582	<0.001
Modified S-ReSC	0.752	0.568–0.995	0.046
CROES	1.033	1.023–1.044	<0.001
Ito’s Nomogram	1.640	1.414–1.902	<0.001
TOHO	0.363	0.252–0.522	<0.001
STONE	0.788	0.635–0.979	0.032
Parenchyma-to-Renal Volume Ratio (RPRV)	1315.9	0.736–2.35 × 10^6^	0.060

**Table 3:** Multivariable logistic regression models of nephrolithometry scores for prediction of uncomplicated single-session stone-free status, adjusted for stone volume, number of stones, previous surgical intervention, and operator experience (≥50 cases).

Scoring System	Adjusted OR Per Point	95% CI	*p*-Value
Ito’s Nomogram	1.697	1.189–2.423	0.004
CROES	1.037	1.012–1.064	0.004
TOHO	0.327	0.141–0.760	0.009
R.I.R.S.	0.371	0.152–0.902	0.029
RUSS	0.264	0.095–0.733	0.011
STONE	0.880	0.541–1.440	0.623
Modified S-ReSC	0.853	0.431–1.689	0.649

Adjusted for stone volume, number of stones, previous surgical intervention, and operator experience ≥50 cases.

**Table 4:** Receiver Operating Characteristic Analysis of Nephrolithometry Scoring Systems and RPRV for Predicting Uncomplicated Single-Session Stone-Free Status.

Variable/Score	AUC	Std. Error	*p* Value *	95% CI
CROES score	0.896	0.024	<0.001	0.850–0.943
Ito’s nomogram	0.862	0.028	<0.001	0.806–0.918
RPRV	0.678	0.042	<0.001	0.595–0.761
Modified S-ReSC score	0.400	0.045	0.031	0.311–0.488
STONE score	0.386	0.045	0.014	0.298–0.474
R.I.R.S. score	0.369	0.044	0.005	0.283–0.455
RUSS score	0.290	0.042	<0.001	0.207–0.372
TOHO score	0.229	0.038	<0.001	0.155–0.303

* Asymptotic significance for null hypothesis AUC = 0.5. For TOHO, RUSS, R.I.R.S., modified S-ReSC, and STONE scores, higher values indicate greater stone complexity and therefore a lower probability of achieving USSSF. Accordingly, AUC values below 0.5 reflect an inverse association with USSSF rather than poor discriminatory performance.

## Data Availability

The data presented in this study are available from the corresponding author upon reasonable request.

## Abbreviations

AUC	Area Under the Receiver Operating Characteristic Curve
BMI	Body Mass Index
CCI	Charlson Comorbidity Index
CI	Confidence Interval
CIRFs	Clinically Insignificant Residual Fragments
CROES	Clinical Research Office of the Endourological Society nephrolithometry score
EAU	European Association of Urology
HU	Hounsfield Unit
IPA	Infundibulopelvic Angle
NCCT	Non-Contrast Computed Tomography
OR	Odds Ratio
PCNL	Percutaneous Nephrolithotomy
RIL	Renal Infundibular Length
RIRS	Retrograde Intrarenal Surgery
R.I.R.S.	R.I.R.S. score
ROC	Receiver Operating Characteristic
RPRV	Renal Parenchyma-to-Renal Volume Ratio
RUSS	Resorlu–Unsal Stone Score
S-ReSC	Seoul National University Renal Stone Complexity
SD	Standard Deviation
STONE	Stone size, Tract length, Obstruction, Number of involved calyces, and Essence (stone density) score
TOHO	T.O.H.O. score
USSSF	Uncomplicated Single-Session Stone-Free Status
